# The Dietetic Treatment of Diabetes

**Published:** 1897-09-25

**Authors:** 


### MEDICAL PROGRESS AND HOSPITAL CLINICS

*The Editor will be glad to receive offers of co-operation and contributions from members of the profession. All letters should be addressed to* The Editor, at the Office, 28 & 29, Southampton Street, Strand, London, W.C.]

### THE DIETETIC TREATMENT OF DIABETES.

The discussion at Montreal on the Dietetic Treatment of Diabetes illustrates very forcibly the reaction which has taken place of late years in regard to the prescription of the strict anti-diabetic diet which was formerly so strictly enforced, to the infinite misery of many patients, although no doubt to the apparent cure of some. Dr. Shingleton Smith, of Bristol, stated, as is undoubtedly true, that patients often prefer a shorter life with a liberal diet to the hope of a longer one with a greatly restricted menu. With the exception of Dr. Duncan, of Glasgow, there seemed to be a general agreement with the lines laid down by Dr. Saundby in opening the discussion.

Dr. Saundby pointed out the futility of persisting in withholding carbohydrates in the hope of removing the last trace of glycosuria. Carbohydrate food was not the only source of sugar. In severe diabetes sugar production and elimination continued, although no doubt much reduced in amount, even when the patient was placed on a flesh diet, a carbohydrate molecule being formed in the process of converting albumin into urea.

Putting physiology on one side, however, clinical experience equally condemned the old routine method of treatment by "anti-diabetic" diet. Experiment must be tried in each case to find out how much carbohydrate the patient could take without injury, or rather what combination of proteid, fat, and carbohydrate gave the best result. To observe the progress of the case the patient should once a week get weighed, and collect and measure the whole of his urine for 24 hours, and a specimen of this urine, being of course an average, should be sent to the doctor for analysis. Of these observations that of the weight was really the most important, for if there was no loss of weight we had the best proof that the diet was right, even though there might be a moderate increase of sugar.

Polyuria and great thirst were best checked by strict diet for a limited time, combined, if necessary, with the administration of a grain or two of extract of opium every night. So far as the details of diet were concerned, the diet he gave might be described as one in which, after a fairly strict regimen for a time, which among other advantages gave a clue to the severity of the case, a gradually increased amount of carbohydrate food was allowed so long as the distressing symptoms remained in abeyance and weight was not lost, but a long time must elapse before ordinary bread was taken, and often the patient had to do without it altogether. The starch contained in potatoes seemed to him to be much less injurious than that in cereals.

